# Bi-stability and critical transitions in mental health care systems: a model-based analysis

**DOI:** 10.1186/s13033-023-00573-y

**Published:** 2023-03-24

**Authors:** Adam Skinner, Jo-An Occhipinti, Ante Prodan, Yun Ju Christine Song, Ian B. Hickie

**Affiliations:** 1 Brain and Mind Centre, Faculty of Medicine and Health, University of Sydney, Sydney, Australia; 2 Computer Simulation and Advanced Research Technologies (CSART), Sydney, Australia; 3 School of Computer, Data and Mathematical Sciences, Western Sydney University, Sydney, Australia

**Keywords:** Australia, Bifurcation, Disease progression, Nonlinear dynamics, Mental health services, System dynamics

## Abstract

**Background:**

Delayed initiation and early discontinuation of treatment due to limited availability and accessibility of services may often result in people with mild or moderate mental disorders developing more severe disorders, leading to an increase in demand for specialised care that would be expected to further restrict service availability and accessibility (due to increased waiting times, higher out-of-pocket costs, etc.).

**Methods:**

We developed a simple system dynamics model of the interaction of specialised services capacity and disease progression to examine the impact of service availability and accessibility on the effectiveness and efficiency of mental health care systems.

**Results:**

Model analysis indicates that, under certain conditions, increasing services capacity can precipitate an abrupt, step-like transition from a state of persistently high unmet need for specialised services to an alternative, stable state in which people presenting for care receive immediate and effective treatment. This qualitative shift in services system functioning results from a ‘virtuous cycle’ in which increasing treatment-dependent recovery among patients with mild to moderate disorders reduces the number of severely ill patients requiring intensive and/or prolonged treatment, effectively ‘releasing’ services capacity that can be used to further reduce the disease progression rate. We present an empirical case study of tertiary-level child and adolescent mental health services in the Australian state of South Australia demonstrating that the conditions under which such critical transitions can occur apply in real-world services systems.

**Conclusions:**

Policy and planning decisions aimed at increasing specialised services capacity have the potential to dramatically increase the effectiveness and efficiency of mental health care systems, promoting long-term sustainability and resilience in the face of future threats to population mental health (e.g., economic crises, natural disasters, global pandemics).

**Supplementary Information:**

The online version contains supplementary material available at 10.1186/s13033-023-00573-y.

## Introduction

Globally, mental disorders accounted for 14.6% of years lived with disability (ranked 2nd, after musculoskeletal disorders) and 4.9% of disability-adjusted life years in 2019 [1], yet the proportion of total government health expenditure allocated to mental health is estimated to be less than 2% [2]. Data from the World Health Organization’s World Mental Health surveys provide evidence for substantial undertreatment of common mental disorders (including depressive, anxiety, and substance use disorders), attributable primarily to low recognition of a need for care and high rates of treatment dropout [3–6]. Thornicroft et al. reported that only 56.7% of people with a major depressive disorder perceive a need for treatment, and among those who receive any care (71.1% of people with a perceived need), only 41.0% receive minimally adequate treatment [5]. Availability and accessibility of mental health services are significant factors affecting treatment initiation among people with a perceived need for care and sustained engagement with services among those who commence treatment; 22.6% of people failing to initiate treatment despite perceiving a need for care and 41.8% of people disengaging from treatment prematurely identify availability and/or accessibility of services as a contributing cause [7].

Delayed initiation and early discontinuation of treatment have potentially significant consequences for the effectiveness and efficiency of mental health care systems. Accumulating evidence indicates that many inadequately treated psychiatric disorders become progressively more severe and persistent with time, increasing the intensity and duration of clinical care required when patients eventually engage (or re-engage) with treatment [8–12]. Any increase in demand for specialised services resulting from higher disorder severity among patients presenting for care would be expected to further restrict service availability and accessibility (due to increased waiting times, higher out-of-pocket costs, etc.), creating a ‘vicious cycle’ that promotes disease progression and undermines the capacity of mental health care systems to provide timely and effective treatment. This paper examines for the first time (at least as far as we are aware) the implications of this positive, or reinforcing, feedback loop for mental health services planning. Using a relatively simple system dynamics model of the interaction of specialised services provision and illness progression, we show via model analysis and an empirical case study that increases in relative services capacity (achieved by increasing the availability and accessibility of services and/or reducing demand for treatment) have the potential to precipitate a critical transition [13] from a state of high unmet need for care to an alternative, stable state in which people engaging with services receive immediate and effective treatment. Our results suggest that policy and planning decisions aimed at expanding specialised services capacity are capable of dramatically increasing not only the effectiveness of mental health care systems, but also their efficiency, promoting resilience and long-term sustainability.

## Methods

### Model structure and assumptions

**Fig. 1:**
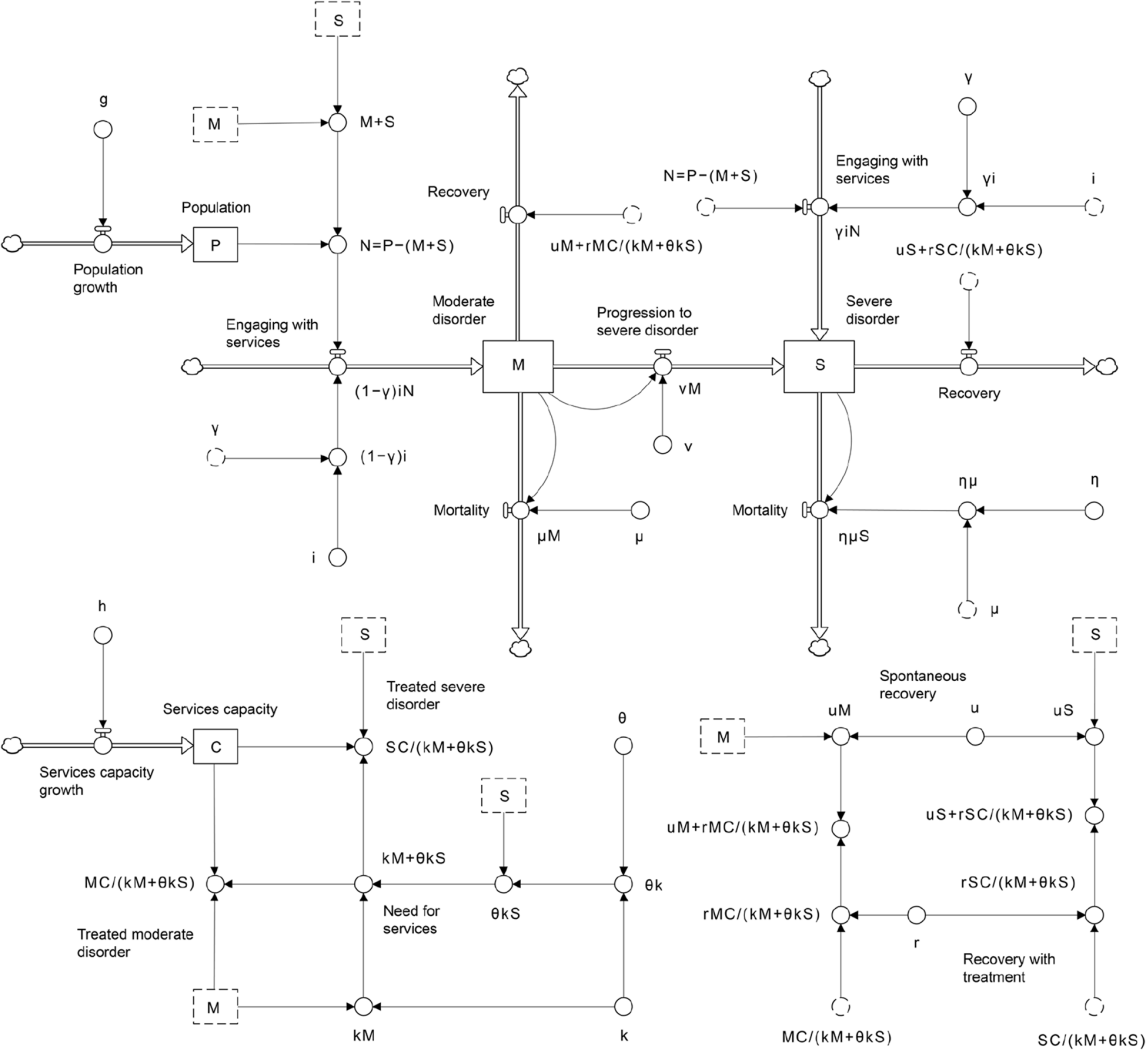
System dynamics model of the interaction of mental health services capacity and disease progression (i.e., the development of severe psychiatric disorders in people with mild to moderate disorders). Symbols are defined in Methods section and Tables 1 and 2

Figure 1 presents the system dynamics model used for the analysis and case study described below (an accessible, public health-focussed introduction to system dynamics modelling is provided in ref. [14]). The core of the model consists of two stocks (i.e., state variables), labelled $M$ and $S$, corresponding to numbers of people with mild or moderate mental disorders ($M$) and severe mental disorders ($S$) who have engaged with mental health services, including those who have obtained a referral from a general practitioner or other medical professional, made an initial appointment with a specialised mental health service provider (e.g., a psychiatrist or a clinical psychologist or other allied health professional), or received any specialised mental health care. People with a perceived need for specialised care who have not yet engaged with services flow into the stocks $M$ and $S$ at rates (per year) equal to $\left(1-\gamma \right)i\left(P-S-M\right)$ and $\gamma i\left(P-S-M\right)$, respectively, where $i$ is the per capita rate at which people engage with specialised services per year, γ is the proportion of people who have a severe disorder when they initially engage with specialised services, and $P$ is the total population. Progression from mild or moderate disorders to severe disorders occurs at a per capita rate $v$, so that $vM$ people with a mild or moderate disorder who have engaged with specialised services develop a severe disorder per year.

People who have engaged with mental health services recover either spontaneously or as a direct result of receiving treatment. Spontaneous recovery occurs at a constant per capita rate $u$, so that $uM$ people with mild or moderate disorders and $uS$ people with severe disorders recover naturally each year (i.e., not as a direct result of receiving specialised care). Treatment-dependent recovery rates for people with mild or moderate disorders and severe disorders are equal to $rMC/\left(kM+\theta kS\right)$ and $rSC/\left(kM+\theta kS\right)$, respectively, where $r$ is the proportion of patients who recover when treated, $C$ is specialised services capacity (the total number of specialised services provided per year), $k$ is the mean number of services required to treat a patient with a mild or moderate disorder, and the ratio $\theta$, equal to the mean number of services required to treat a patient with a severe disorder divided by $k$, is assumed to be greater than 1 (i.e., patients with severe disorders are assumed to require more services than those with mild or moderate disorders). Note that as the number of people with severe disorders engaged with specialised services increases, the total number of people receiving treatment per year, $C\left(M+S\right)/\left(kM+\theta kS\right)$, and the proportion of patients treated who have mild or moderate disorders, $M/\left(M+S\right)$, decline (assuming $M$ and $S$ are positive).

Per capita mortality rates for people with mild or moderate and severe mental disorders are equal to $\mu$ and $\eta \mu$, respectively, where we assume $\eta$ is greater than 1 (i.e., the per capita mortality rate for people with severe mental disorders is assumed to be higher than that for people with mild or moderate disorders). People are added to the total population ($P$) through births and migration at a constant rate $g$ per year. For simplicity, we assume that the numbers of people in the population with mild or moderate and severe mental disorders who have engaged with services are not directly affected by migration; thus, people added to the population are initially not engaged with specialised care, while those who have engaged with services only leave the stocks $M$ and $S$ through recovery, mortality, or disease progression (see Fig. 1). Specialised mental health services capacity, $C$, is assumed to increase at a constant yearly rate of $h$ services per year. The population and services capacity growth rates, $g$ and $h$, were set to zero for the model analysis described below (i.e., we assumed a constant total population and constant services capacity; see Table 1); for the case study, $g$ and $h$ were estimated from empirical data, as described in Additional file 1: Appendix S1.

**Table 1:** Parameter values assumed in the model analysis

Parameter	Symbol	Value	Reference(s)
Initial number of people with mild to moderate disorders engaged with services	*M*_0_	–	
Initial number of people with severe disorders engaged with services	*S*_0_	–	
Initial population	*P*_0_	1,000,000	
Population increase per year	*g*	0	
Per capita services engagement rate	*i*	0.05496	[15, 16]
Proportion of people with a severe disorder when they initially engage with services	*γ*	0	
Per capita disease progression rate	*v*	0.5882	[15, 16]
Per capita spontaneous recovery rate	*u*	0.3096	[34]
Initial services capacity (services per year)	*C*_0_	–	
Services capacity increase per year	*h*	0	
Services required per patient (mild to moderate disorders)	*k*	2	
Ratio of services required for patients with severe disorders versus patients with mild to moderate disorders	*θ*	10	
Proportion of patients recovering when treated	*r*	0.4460	[35]
Per capita mortality rate (mild to moderate disorders)	*μ*	0.008772	[36]
Mortality hazard ratio (severe disorders)	*η*	1.3971	[37]

### Model analysis

The potential impacts of modifying service availability and accessibility on the effectiveness and efficiency of mental health care systems were assessed by determining equilibrium numbers of people with mild or moderate and severe mental disorders engaged with specialised services over a range of values for the services capacity parameter $C$. At equilibrium, the numbers of people entering and leaving the stocks $M$ and $S$ per year are equal, so the numbers of people with mild or moderate and severe disorders engaged with specialised services remain constant. Any particular equilibrium may be stable or unstable; the state of a system (here, the values of the stocks $M$ and $S$) that is displaced from an equilibrium (e.g., by an abrupt change in services capacity that alters treatment-dependent recovery rates) will tend to move towards a stable equilibrium and away from an unstable equilibrium over time. Our principal aim in the analyses presented here was to examine changes in the number of equilibria and the potential for abrupt transitions between alternative stable states as services capacity (a measure of the availability and accessibility of mental health services) is altered.

Equilibrium numbers of people with mild or moderate and severe disorders engaged with specialised services were calculated assuming the parameter values in Table 1 (formulas yielding the equilibrium values of the stocks $M$ and $S$ for a given set of parameter values are derived in Additional file 1: Appendix S2). The values specified for the per capita rate at which people engage with services, $i$, and the per capita rate of progression from mild or moderate to severe disorders, $v$, yield equilibrium values for $M$ and $S$ equal to 4.7% and 6.2% of the total population, respectively, consistent with the proportions of adults (aged 18 years and above) with moderate psychological distress (Kessler 10 [K10] scores 16 − 21) and high to very high psychological distress (K10 scores 22 or more) who perceived a need for services in Australia over the period 2007 − 2018 [15, 16]. We examined the effects of modifying $i$, $v$, the proportion of people with a severe disorder when they initially engage with services ($\gamma$), the proportion of patients recovering with treatment ($r$), and the ratio $\theta$ (i.e., the number of services required per patient to treat severe disorders versus mild to moderate disorders) by varying each parameter independently (non-focal parameters were fixed at the values in Table 1). The (local) stability of each equilibrium was determined via linear stability analysis, as described in Additional file 1: Appendix S2.

### Case study: child and adolescent mental health services in Australia

The model analysis described above enables us to identify the specific conditions under which we would expect changes in specialised services capacity to precipitate an abrupt shift in the effectiveness and efficiency of mental health care systems. Whether these conditions apply in real services systems is an empirical question that we address here through a detailed case study of child and adolescent mental health services (CAMHS) in the Australian state of South Australia. Public (i.e., government-funded) CAMHS in Australia provide tertiary-level, community-based care to children and adolescents (aged 0 − 17 years) presenting with complex psychiatric conditions, typically characterised by higher disorder severity (e.g., psychotic disorders, personality disorders, treatment-resistant depressive disorders, suicidal ideation, deliberate self-harm) and/or multiple adverse environmental exposures (parental separation, family violence, socio-economic deprivation, parental substance abuse, etc.). Segal et al. estimated that in 2016 − 17, the cost of delivering specialised mental health care, including assessment, clinical management, psychiatric oversight, and psychosocial support, to the *c*. 27 thousand South Australian children and adolescents with tertiary-level needs (*c*. 7.3% of the population aged 0 − 17 years) was 5.3 times actual government expenditure (AU$127 million versus AU$24 million), suggesting that access to CAMHS in South Australia is potentially severely limited by capacity constraints [17].

Bayesian Markov chain Monte Carlo (MCMC) methods [18] were used to fit the system dynamics model in Fig. 1 to estimates of the prevalence of severe mental health conditions and moderate mental health conditions among South Australian children and adolescents (0 − 17 years) for the period 1 January 2017 to 30 June 2019 (prevalence estimates were adjusted for perceived need for specialised care; see Additional file 1: Appendix S3). Letting ${y}_{j}\left(t\right)$ and ${m}_{j}\left(t,\phi \right)$ denote, respectively, the observed value of time series $j$ at time $t$ (e.g., the prevalence of severe mental disorders in 2018) and the corresponding simulation model output (the modelled prevalence of severe disorders in 2018) obtained for a particular set of parameter values $\phi$, the likelihood for each ${y}_{j}\left(t\right)$ was calculated as

$$p\left({y}_{j}\left(t\right)\mid\phi ,{\alpha }_{j}\right)=Beta\left({y}_{j}\left(t\right)\mid{\alpha }_{j},\frac{{\alpha }_{j}\left(1-{m}_{j}\left(t,\phi \right)\right)}{{m}_{j}\left(t,\phi \right)}\right)\text{,}$$

i.e., we assumed that the observed prevalence values, ${y}_{j}\left(t\right)$, follow a beta distribution with mean ${m}_{j}\left(t,\phi \right)$. The parameter vector $\phi$ contains the per capita rate at which people engage with specialised services ($i$), the per capita rate of progression from moderate to severe disorders ($v$), the initial prevalence of severe disorders (i.e., the value of $S/P$ at the start of 2017), and the initial prevalence of moderate disorders ($M/P$ at the start of 2017); values for the remaining dynamic model parameters were derived from published data (see Table 2). Deviations of the observed data from their expected value were assumed to be independent, so the likelihood for the combined time series data (i.e., prevalence estimates for moderate disorders and severe disorders) is the product of the likelihoods for all ${y}_{j}\left(t\right)$. Posterior simulation was performed using Stan ver. 2.21.2 (see Additional file 1: Appendix S3) [19].

**Table 2:** Parameter values and prior distributions specified in the case study of tertiary-level child and adolescent mental health services (CAMHS) in South Australia

Parameter	Symbol	Value	Reference(s)
Initial proportion of people with a moderate disorder and a perceived need for services	*M*_0_/*P*_0_	normal(0.04024, 0.001006) T[0, 1]^†﻿^	[17, 38]
Initial proportion of people with a severe disorder and a perceived need for services	*S*_0_/*P*_0_	normal(0.02941, 0.0007352) T[0, 1]	[17, 38]
Initial population	*P*_0_	364622.2364	See Additional file 1: Appendix S1
Population increase per year	*g*	1823.6364	See Additional file 1: Appendix S1
Per capita services engagement rate	*i*	normal (0, 0.1) T[0,]	
Proportion of people with a severe disorder when they initially engage with services	*γ*	0.03468	[38]
Per capita disease progression rate	*v*	normal(0, 0.1) T[0,]	
Per capita ageing rate^‡^	*u*	0.05556 (i.e., 1/18)	
Initial services capacity (services per year)	*C*_0_	68773.7817	See Additional file 1: Appendix S1
Services capacity increase per year	*h*	970.2012	See Additional file 1: Appendix S1
Services required per patient (moderate disorders)	*k*	1.9423	[39]
Ratio of services required for patients with severe disorders versus patients with moderate disorders	*θ*	9.3923	[39]
Proportion of patients recovering when treated	*r*	0.6029	[40]
Per capita mortality rate (moderate disorders)	*μ*	0.0004225	[36]
Mortality hazard ratio (severe disorders)	*η*	1.2190	[37]

^†^Prior distributions are given using Stan notation (see ref. [19])

^‡^Note that the per capita spontaneous recovery rate is assumed to equal 0; the parameter *u* is used to model outflows from the stocks *M* and *S* due to ageing (i.e., adolescents turning 18)

## Results

### Model analysis

**Fig. 2:**
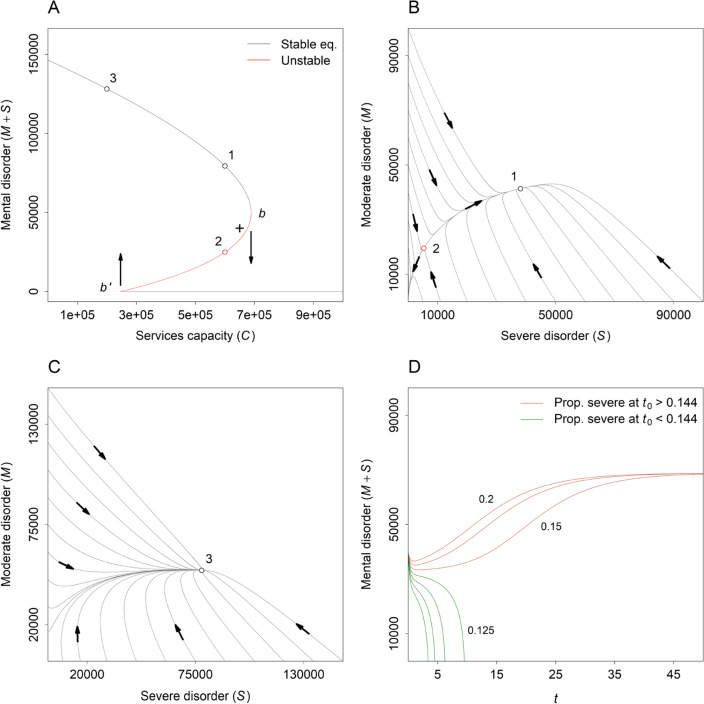
Model analysis results. **A** Bifurcation diagram showing equilibrium numbers of people engaged with mental health services over a range of values of the services capacity parameter $C$. See Results section for a detailed description (see also ref. [13] for an accessible introduction to bifurcation diagrams and their interpretation). The numbered equilibrium points (1 − 3) correspond to those in panels **B** and **C**. The cross indicates the initial position of the system in the simulations shown in panel **D** (i.e., when $t$ is equal to 0). **B** Phase plane plot showing simulated trajectories for the numbers of people with mild to moderate ($M$) and severe ($S$) disorders engaged with services when $C$ is equal to 600 thousand services per year. **C** Phase plane plot showing trajectories for $M$ and $S$ when $C$ is equal to 200 thousand services per year. **D** Simulations showing the effect of the proportion of people engaged with services who have a severe disorder on unmet need for specialised care (i.e., $M+S$) at equilibrium. Services capacity and the initial level of unmet need are identical in all simulations (see panel **A**). When the initial proportion of people engaged with services who have a severe disorder exceeds *c*. 0.144, the system evolves towards an equilibrium state of high unmet need for specialised care; when this proportion is less than *c*. 0.144, the number of people engaged with services declines rapidly to near zero

Panel A in Fig. 2 presents equilibrium numbers of people with mild to moderate or severe mental disorders engaged with specialised services over a range of values of the services capacity parameter, $C$. For values of $C$ less than 246,462 services per year, there is a single, stable equilibrium, at which more than *c*. 120 thousand people (i.e., 12% of the total population) are engaged with services at any point in time (see Fig. 2, panel C). As capacity increases (moving along the upper part of the curve from equilibrium point 3), the equilibrium number of people engaged with mental health services declines gradually until $C$ reaches 688,619 services per year. Past this point ($b$ in Fig. 2, panel A), treatment-dependent recovery among people with mild to moderate disorders reduces the disease progression rate (i.e., $pM$) sufficiently to sustain a reinforcing feedback loop in which the number of patients treated per year increases as the rate at which people develop severe disorders after engaging with services declines, further increasing treatment-dependent recovery among people with mild to moderate disorders. This ‘virtuous cycle’ rapidly drives the number of people engaged with specialised services to near zero, at which point people are being treated immediately and recovering as they engage with the mental health care system.

As the number of people with severe disorders engaged with services approaches zero, the number of patients that can be treated per year approaches $C/k$ (i.e., the total number of services provided per year divided by the number of services required per patient to treat mild to moderate disorders). Thus, above the threshold value of $C$ at which the number of people engaged with services declines abruptly towards zero (688,619 services per year; see above), there is sufficient capacity to treat at least *c*. 344 thousand people per year (i.e., assuming $S$ is near zero), which is more than 6 times the maximum service engagement rate (equal to $iP$, or *c*. 55 thousand people per year). Accordingly, once the number of people engaged with specialised services has declined to near zero, capacity can be reduced significantly without affecting the ability of services to provide immediate and effective treatment to everyone presenting for care. The number of people engaged with services only begins to increase when $C$ declines to less than 246,462 services per year ($b{^{\prime}}$ in Fig. 2, panel A). At this point, the rate at which people develop severe disorders after engaging with services also increases, which reduces the number of patients with mild to moderate disorders treated per year, further increasing the disease progression rate. The result is an abrupt increase in the number of people engaged with services to the stable equilibrium on the upper part of the curve in panel A of Fig. 2. Between the threshold capacity values (or bifurcation points) [20] at $b{^{\prime}}$ and $b$, the system may evolve towards either of the alternative stable states (corresponding to high unmet need for specialised care or no unmet need) depending on available services capacity and the initial values of the stocks $M$ and $S$ (Fig. 2, panels B and D).

**Fig. 3:**
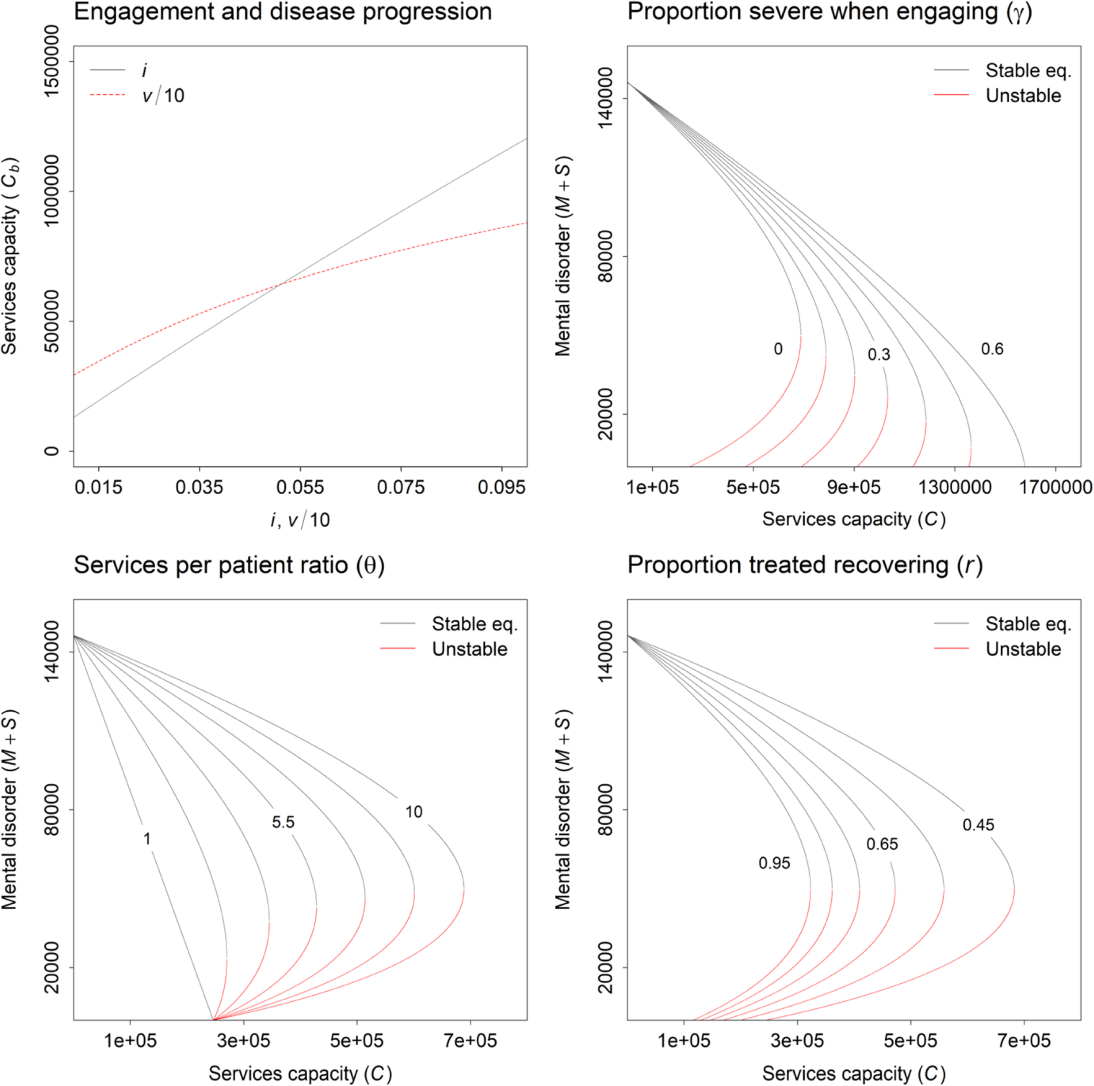
Effects of independently varying the values of selected model parameters on the equilibrium numbers of people engaged with mental health services and the critical capacity ${C}_{b}$ above which the services system transitions from a state of high unmet need for care to a state of no unmet need (i.e., the value of $C$ at $b$ in Fig. 2, panel **A**)

Figure 3 shows the effects of independently varying the values of selected model parameters on the equilibrium numbers of people engaged with mental health services. The critical capacity ${C}_{b}$ above which the services system transitions from a state of high unmet need for care to a state of no unmet need (i.e., the value of $C$ at $b$ in Fig. 2, panel A) increases as the per capita rate at which people engage with services ($i$) and the per capita rate of progression from mild or moderate to severe disorders ($v$) increase (since more people engage with services and develop severe disorders per year). ${C}_{b}$ also increases as the proportion of people who have severe disorders when they initially engage with services ($\gamma$) increases from zero up to *c*. 0.5. Above this point (i.e., when $\gamma$ > *c*. 0.5), the ability of services to reduce demand for care by preventing disease progression is insufficient to precipitate an abrupt transition via the feedback mechanism described above, and the equilibrium number of people engaged with services approaches zero gradually with increasing $C$. As the proportion of people recovering when treated ($r$) increases, the number of services required to successfully treat patients presenting for care declines, reducing ${C}_{b}$, while increasing the ratio of the mean numbers of services needed to treat patients with mild to moderate disorders and severe disorders ($\theta$) increases ${C}_{b}$, since the number of services required per patient to treat severe disorders increases (note that when $\theta$ is equal to 1, reducing the number of people developing severe disorders after engaging with services does not affect the number of patients treated per year; thus, the feedback loop generating alternative stable states is effectively disabled, and there is only one stable equilibrium for each value of $C$).

### Case study

**Fig. 4:**
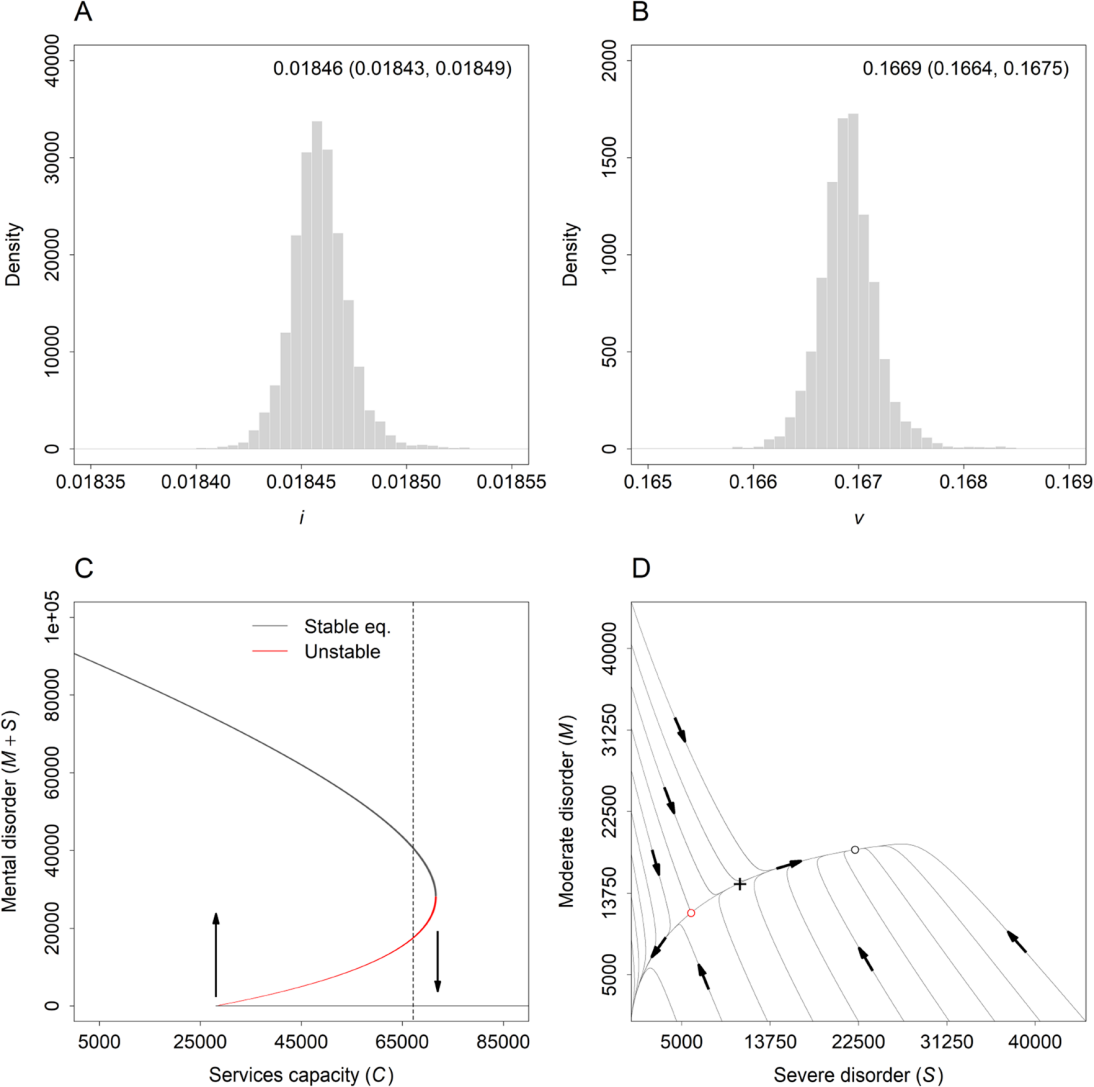
Case study results. **A**, **B** Marginal posterior distributions for the per capita rate at which children and adolescents engage with tertiary-level mental health services ($i$) and the per capita rate of progression from moderate to severe psychiatric conditions ($v$). Median estimates and 95% posterior intervals are shown in the top right corner of each panel. Prior distributions are plotted as smooth curves. **C** Bifurcation diagram showing equilibrium numbers of children and adolescents engaged with tertiary-level services over a range of values of the services capacity parameter $C$ (see Results section for details). The dashed vertical line corresponds to the number of services provided in 2017 (67,156). **D** Phase plane plot showing trajectories for the numbers of children and adolescents with moderate and severe disorders engaged with tertiary-level services, simulated assuming a value of 67,156 services per year for $C$ (i.e., the number of services provided in 2017) and median estimates for the per capita rates $i$ and $v$ derived from the Markov chain Monte Carlo (MCMC) analysis (see panels **A** and **B**). Estimated numbers of South Australian children and adolescents with tertiary-level needs in 2017 are indicated with a cross. Panels **C** and **D** were generated assuming a total population of 365,965 children and adolescents (the estimated number of South Australian residents aged 0 − 17 years in mid-2017)

Marginal posterior distributions for the per capita rate at which South Australian children and adolescents with moderate or severe mental disorders engage with tertiary-level CAMHS ($i$) and the per capita rate of progression from moderate to severe disorders ($v$) are presented in Fig. 4 (panels A and B). Panel C in Fig. 4 shows equilibrium numbers of children and adolescents engaged with CAMHS calculated for each of 100 randomly chosen parameter vectors ($\phi$) sampled in the MCMC analysis. Assuming median estimates for $i$ and $v$ based on all MCMC samples (Fig. 4, panels A and B), the bifurcation points corresponding to those at $b{^{\prime}}$ and $b$ in Fig. 2 (panel A) are equal to 28,097 services per year and 71,616 services per year, respectively; thus, unmet need for tertiary-level care declines abruptly to zero when services capacity exceeds *c*. 72 thousand services per year. A total of 67,156 service contacts were recorded by CAMHS in South Australia in 2017 [17], or 93.8% of the estimated number of contacts required to precipitate a critical transition to a stable state in which all children and adolescents with tertiary-level needs receive immediate and effective treatment (Fig. 4, panels C and D).

## Discussion

The model analysis and case study presented here indicate that the availability and accessibility of specialised services may have a profound impact on the effectiveness and efficiency of mental health care systems. Where the rate of specialised services provision is insufficient to ensure that people engaging with services receive timely and effective treatment (due to high out-of-pocket costs, workforce-related capacity constraints, sub-optimal distribution of services, etc.), many people with mild to moderate disorders may be expected to develop more severe disorders, leading to an increase in demand for mental health care that further reduces service availability and accessibility. As the capacity of specialised services to adequately treat people presenting for care increases, treatment-dependent recovery among patients with mild to moderate disorders increases, reducing the rate of disease progression. The resulting decrease in the number of severely ill patients requiring intensive and/or prolonged treatment effectively ‘releases’ capacity that can be used to further reduce the development of severe disorders among people engaged with the services system. Under certain conditions (e.g., where the proportion of people who have a severe disorder when they first engage with services is relatively low; Fig. 3), this reinforcing feedback loop may be strong enough to precipitate an abrupt transition from a state of high unmet need for services to an alternative stable state in which everyone presenting for care receives immediate and effective treatment. Our empirical case study of tertiary-level CAMHS in South Australia provides evidence that the conditions under which such critical transitions can occur apply in at least some services systems.

Precipitating a qualitative shift in services system functioning (from a state of persistently high unmet need for specialised care to a state of no unmet need) via an expansion of services capacity may in many cases depend on a substantial increase in the number of specialised mental health professionals providing clinical care (psychiatrists, clinical psychologists, etc.). However, policies aimed at reducing deficits in clinical workforce capacity would generally be expected to have minimal near-term impact on the services provision rate (due to training lead times) [21], so that additional interventions designed to reduce overall demand for care may often be necessary to address immediate capacity constraints. Our analyses identify several possible means of reducing the number of people engaged with specialised services where services capacity is fixed (see Fig. 3), including: 1) increasing the proportion of people engaging with services before they develop severe disorders (e.g., by lowering the financial cost of accessing treatment, improving screening for mental disorders in primary care); 2) reducing engagement and disease progression rates via modification of population-level exposure to established risk factors for psychological distress and associated mental disorders (e.g., unemployment, poverty, debt, homelessness) [22]; and 3) increasing treatment-dependent recovery by improving the effectiveness of existing services (e.g., via the routine use of technology to provide better co-ordinated and more personalised, measurement-based care) [23]. Policy approaches combining measures for increasing services capacity (moving the services system to the right in Fig. 2, panel A) and reducing overall demand for specialised care (shifting the bifurcation point at $b$ to the left) may be assumed to hold the greatest potential for generating an abrupt decline in unmet need for treatment.

The case study results presented in Fig. 4 (panel C) indicate that increasing the capacity of CAMHS in South Australia by 6.6% (from 67,156 to 71,616 service contacts per year) would be sufficient to produce an abrupt transition to a state in which children and adolescents presenting with tertiary-level needs receive immediate and effective treatment (note, however, that this transition is more rapid for larger capacity increases; see Additional file 1: Appendix S4). This estimate is dramatically lower than the more than five-fold increase in capacity Segal et al. estimated would be necessary to provide adequate care to all South Australian children and adolescents with complex psychiatric conditions [17]. Partly, this discrepancy is attributable to the fact that Segal et al.’s needs-based workforce model is static rather than dynamic (tertiary-level need is assumed to remain constant), effectively prohibiting a decline in demand for services as patients are treated and recover [17]. As our model analysis demonstrates, treatment-dependent recovery impacts substantially on the services provision rate required to achieve complete treatment coverage for people with a need for specialised care. Any increase in the recovery rate resulting from an expansion of treatment capacity directly reduces the number of people engaged with services, reducing not only current demand for care, but also the disease progression rate (which depends on $M$). As a consequence, the proportion of people engaged with services who have a severe mental disorder, and thus the mean number of services required per patient, decreases (see Additional file 1: Appendix S2), further increasing the recovery rate. Demand for services therefore declines at an increasing rate as capacity is increased, so that Segal et al.’s assumption of constant need will generally not hold.

### Limitations

Several important determinants of the effectiveness and efficiency of mental health care systems, including the mix of service types, the distribution of service providers relative to need, and the level of co-ordination across services [24–26], were not modelled explicitly in the analyses presented here. Additionally, our analyses effectively disregard potentially significant heterogeneity in individual-level factors affecting service engagement (i.e., help-seeking), disease progression, and recovery rates (principal diagnosis, comorbidities, age, gender, etc.) and assume that the maximum per capita recovery rate is constrained only by specialised services capacity. Analyses of more complex models of the interaction of mental health services provision and disease progression are needed to determine the extent to which our conclusions depend on these assumptions [27, 28]. More generally, our analytical approach focusses on identifying stable equilibrium states, since we expect a system to converge to these states over time, yet convergence can be relatively slow, and systems may often be far from a stable equilibrium (e.g., when an equilibrium is moving continuously due to population growth, changing services capacity, etc.). The critical transitions in services system functioning described here are abrupt in the sense that a very small increase in capacity above the threshold value ${C}_{b}$ causes the equilibrium number of people engaged with specialised services to suddenly ‘jump’ to a substantially lower value; however, the actual number of people engaged with services approaches this equilibrium gradually, potentially over an extended period of time (see Additional file 1: Appendix S4).

## Conclusion

The substantial underinvestment in specialised services highlighted by the World Health Organization’s *Mental Health Atlas* project has potentially significant consequences for the functioning of mental health care systems worldwide [2]. Delayed initiation and early discontinuation of treatment due to limited availability and accessibility of services may, in many cases, result in people with mild or moderate disorders developing more severe disorders, leading to an increase in demand for specialised care that exacerbates existing capacity constraints. Policy and planning decisions aimed at increasing relative services capacity via either an increase in the services provision rate or a decrease in demand for care have the potential not only to directly increase treatment-dependent recovery, but also to prevent the development of severe disorders in patients with mild to moderate disorders [29]. The model analysis and case study presented here indicate that a reduction in the disease progression rate among people engaged with specialised services may precipitate an abrupt transition from a state of persistently high unmet need for care to an alternative state in which people with a perceived need for services receive immediate and effective treatment. This abrupt transition is accompanied by a decline in the number of services required per patient (as the proportion of patients with severe disorders declines), so that strategic investment in capacity expansion may be capable of transforming not only the effectiveness of mental health care systems, but also their efficiency, promoting long-term sustainability and resilience in the face of inevitable future threats to population mental health (e.g., economic crises, natural disasters, global pandemics) [30–33].

## Supplementary Information

**Additional file 1.** Additional appendices.

## Data Availability

The datasets used and/or analysed during the current study are available from the corresponding author on reasonable request.

## Abbreviations

CAMHS: Child and adolescent mental health services
K10: Kessler 10
MCMC: Markov chain Monte Carlo
