# Supplementary material for: Bi-stability and critical transitions in mental health care systems: a model-based analysis
Source: Int J Ment Health Syst. 2023 Mar 24;17:5. doi: 10.1186/s13033-023-00573-y (PMC10037813; doi:10.1186/s13033-023-00573-y)
Supplement: Supplementary file 1 — Additional file 1. Additional appendices. [file 13033_2023_573_MOESM1_ESM.docx]

Supplementary information. Bi-stability and critical transitions in mental health care systems.

Adam Skinner^1,*^, Jo-An Occhipinti^1,2^, Ante Prodan^2,3^, Yun Ju Christine Song^1^, Ian B. Hickie^1^

^1^ Brain and Mind Centre, Faculty of Medicine and Health, University of Sydney, Sydney, Australia; ^2^ Computer Simulation and Advanced Research Technologies (CSART), Sydney, Australia; ^3^ School of Computer, Data and Mathematical Sciences, Western Sydney University, Sydney, Australia

^*^ Corresponding author. Email: adam.skinner@sydney.edu.au. Address: Level 4, Building M02C, 94 Mallet Street, Camperdown, NSW 2050, Australia. Phone: +61 450 458 201.

Supplementary appendix 1

Estimation of population and services capacity parameters for the case study

Estimates of the initial values of the population and services capacity stocks $P$ and $C$ (i.e., at the start of 2017) and the rates of population and services capacity growth per year ($g$ and $h$) specified in the case study (see Table 2 of the paper) were obtained via simple linear regression, using population estimates published by the Australian Bureau of Statistics (2021) and data on state-financed community mental health care services provision derived from Segal et al. (2018) and the Australian Institute of Health and Welfare (2021) (see figure S1).


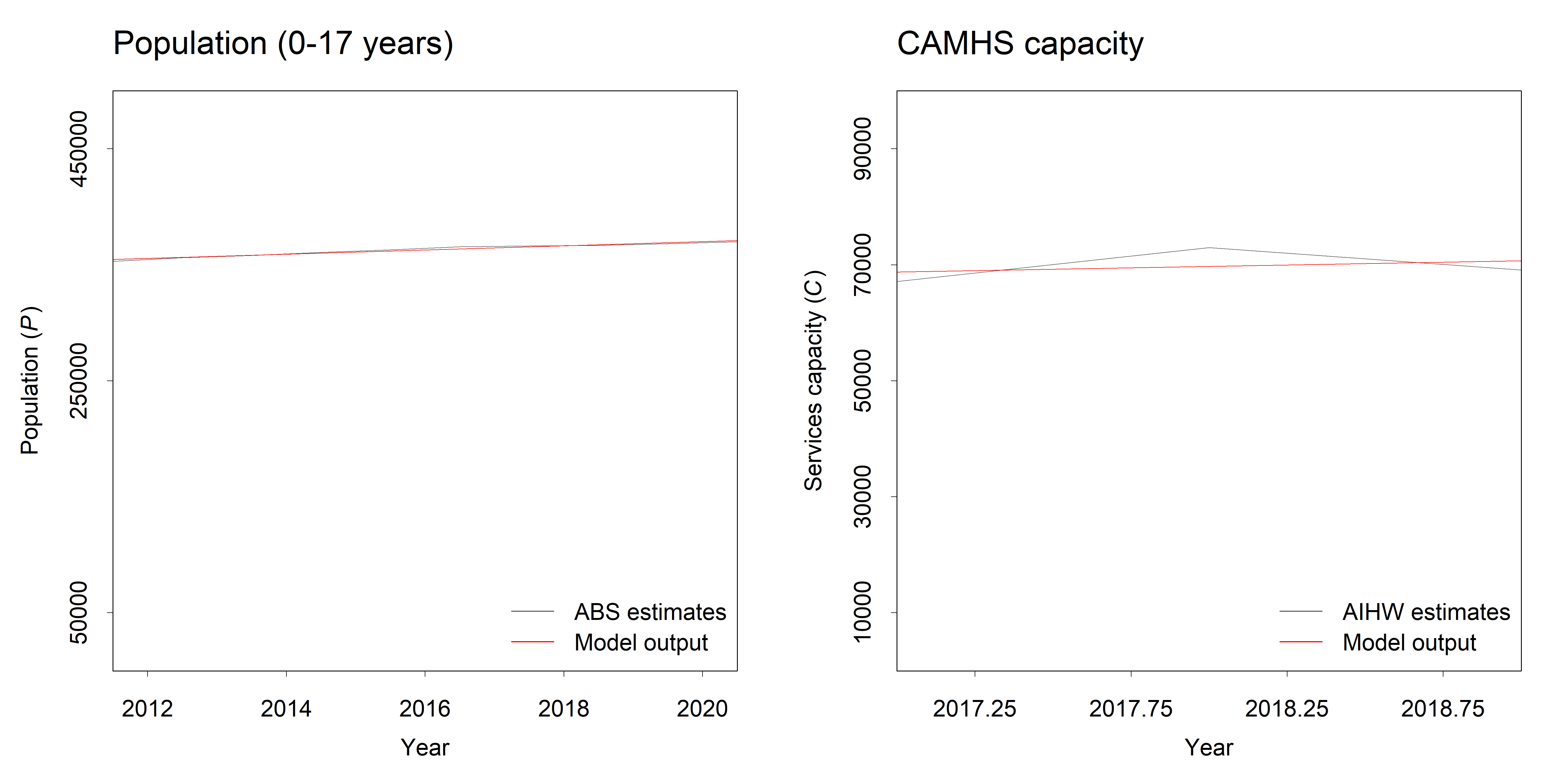


Figure S1. Population and child and adolescent mental health services (CAMHS) capacity estimates derived from the model and from data published by the Australian Bureau of Statistics (ABS) and the Australian Institute of Health and Welfare (AIHW).

References

Australian Bureau of Statistics, 2021. National, state and territory population, Dec 2020. Cat. no. 3101.0. Australian Bureau of Statistics, Canberra.

Australian Institute of Health and Welfare, 2021. Mental health services in Australia. State and territory community mental health care 2018−19. Available at: https://www.aihw.gov.au/reports/mental-health-services/mental-health-services-in-australia/data.

Segal, L., Guy, S., Leach, M., Groves, A., Turnbull, C., Furber, G., 2018. A needs-based workforce model to deliver tertiary-level community mental health care for distressed infants, children, and adolescents in South Australia: a mixed-methods study. Lancet Public Health 3, e296‒303.

Supplementary appendix 2

Derivation of the equilibrium numbers of people with mild to moderate and severe disorders engaged with specialised services (and other analytical results)

*2.1. Derivation of the equilibrium numbers of people engaged with services*

At equilibrium, the numbers of people with mild to moderate and severe disorders engaged with specialised services ($M$ and $S$, respectively) remain constant, i.e., their rates of change are equal to 0, so we have the equilibrium conditions

$$\frac{dM}{dt}=\left( 1-\gamma\right)i\left( P-M^{*}-S^{*} \right)-M^{*}\left( v+\mu+u \right)-\frac{rM^{*}C}{kM^{*}+\theta kS^{*}}=0$$

$$\frac{dS}{dt}=\gamma i\left( P-M^{*}-S^{*} \right)+vM^{*}-S^{*}\left( \eta\mu+u \right)-\frac{rS^{*}C}{kM^{*}+\theta kS^{*}}=0\text{,}$$

where $t$ is time in years, $M^{*}$ and $S^{*}$ are the equilibrium numbers of people with mild to moderate and severe disorders engaged with services, and all other notation is the same as in figure 1 of the paper. Note that the total population ($P$) and services capacity ($C$) are assumed to be constant (see Methods section of the paper). Services capacity, $C$, is the total number of specialised services provided to patients with mild to moderate and severe disorders per year. The number of services provided to patients with severe disorders per year is ${\theta kSC}/\left( kM+\theta kS \right)$, i.e., the proportion of total current demand for specialised care, $kM+\theta kS$, attributable to people with severe disorders multiplied by the number of services provided per year (i.e., specialised services capacity, $C$). The number of services provided to patients with mild to moderate disorders per year, ${kMC}/\left( kM+\theta kS \right)$, is therefore equal to $C-{\theta kSC}/\left( kM+\theta kS \right)$, so that the first equilibrium condition above can be expressed as

$$\frac{dM}{dt}=\left( 1-\gamma\right)i\left( P-M^{*}-S^{*} \right)-M^{*}\left( v+\mu+u \right)-\frac{r}{k}\left( C-\frac{\theta kS^{*}C}{kM^{*}+\theta kS^{*}} \right)$$

$$=\left( 1-\gamma\right)i\left( P-M^{*}-S^{*} \right)-M^{*}\left( v+\mu+u \right)-\frac{rC}{k}+\theta\frac{rS^{*}C}{kM^{*}+\theta kS^{*}}=0\text{.}$$

From the second equilibrium condition, we have

$$\frac{rS^{*}C}{kM^{*}+\theta kS^{*}}=\gamma i\left( P-M^{*}-S^{*} \right)+vM^{*}-S^{*}\left( \eta\mu+u \right)\text{,}$$

so, at equilibrium

$$\frac{dM}{dt}=\left( 1-\gamma\right)i\left( P-M^{*}-S^{*} \right)-M^{*}\left( v+\mu+u \right)-\frac{rC}{k}+\theta\left[ \gamma i\left( P-M^{*}-S^{*} \right)+vM^{*}-S^{*}\left( \eta\mu+u \right) \right]=0\text{.}$$

Solving for $S^{*}$, we obtain

$$S^{*}=\frac{P\left( i-\gamma i+\theta\gamma i \right)-M^{*}\left[ \left( 1-\gamma\right)i+\theta\left( \gamma i-v \right)+v+\mu+u \right]-{rC}/k}{i-\gamma i+\theta\left( \gamma i+\eta\mu+u \right)}\text{.}$$

Substituting the solution for $S^{*}$ above into the equilibrium condition ${dM}/{dt}=\left( 1-\gamma\right)i\left( P-M^{*}-S^{*} \right)-M^{*}\left( v+\mu+u \right)-{rM^{*}C}/\left( kM^{*}+\theta kS^{*} \right)=0$ and simplifying yields the quadratic equation $a_{2}\left( M^{*} \right)^{2}+a_{1}M^{*}+a_{0}=0$, where

$$a_{2}=k\left[ i-\gamma i-\theta\left( i-2\gamma i+\theta\left( \gamma i-v \right)+v+\mu-\eta\mu\right) \right]\left\{ \frac{\left( 1-\gamma\right)i\left[ \left( 1-\gamma\right)i+\theta\left( \gamma i-v \right)+v+\mu+u \right]}{i-\gamma i+\theta\left( \gamma i+\eta\mu+u \right)}-\left( 1-\gamma\right)i-v-\mu-u \right\}$$

$$a_{1}=k\left[ i-\gamma i-\theta\left( i-2\gamma i+\theta\left( \gamma i-v \right)+v+\mu-\eta\mu\right) \right]\left\{ \left( 1-\gamma\right)iP-\frac{\left( 1-\gamma\right)i\left[ P\left( i-\gamma i+\theta\gamma i \right)-{rC}/k \right]}{i-\gamma i+\theta\left( \gamma i+\eta\mu+u \right)} \right\}+\theta\left[ kP\left( i-\gamma i+\theta\gamma i \right)-rC \right]\left\{ \frac{\left( 1-\gamma\right)i\left[ \left( 1-\gamma\right)i+\theta\left( \gamma i-v \right)+v+\mu+u \right]}{i-\gamma i+\theta\left( \gamma i+\eta\mu+u \right)}-\left( 1-\gamma\right)i-v-\mu-u \right\}-rC\left[ i-\gamma i+\theta\left( \gamma i+\eta\mu+u \right) \right]$$

$$a_{0}=\theta\left[ kP\left( i-\gamma i+\theta\gamma i \right)-rC \right]\left\{ \left( 1-\gamma\right)iP-\frac{\left( 1-\gamma\right)i\left[ P\left( i-\gamma i+\theta\gamma i \right)-{rC}/k \right]}{i-\gamma i+\theta\left( \gamma i+\eta\mu+u \right)} \right\}\text{.}$$

Solving $a_{2}\left( M^{*} \right)^{2}+a_{1}M^{*}+a_{0}=0$ for $M^{*}$ using the quadratic formula yields two solutions for the equilibrium number of people with mild to moderate disorders engaged with specialised services

$$M^{*}=\frac{-a_{1}\pm\sqrt{{a_{1}}^{2}-4a_{2}a_{0}}}{2a_{2}}\text{,}$$

which can be substituted into the solution for $S^{*}$ above to obtain the corresponding equilibrium numbers of people with severe disorders engaged with specialised services. These two solutions are the high unmet need and unstable equilibria in figures 2−4 of the paper (the equilibrium points labelled 1 and 2 in figure 2, for example, are obtained using the above formulas for $M^{*}$ and $S^{*}$ when $C$ is equal to 600 thousand services per year).

Note that ${dM}/{dt}$ and ${dS}/{dt}$ are undefined when $M=S=0$, as the denominators in the treatment-dependent recovery rate terms ${rMC}/\left( kM+\theta kS \right)$ and ${rSC}/\left( kM+\theta kS \right)$ are zero. However, when $M$ and $S$ are equal to zero, treatment-dependent recovery per year must also equal zero, so that ${dM}/{dt}=\left( 1-\gamma\right)iP$ and ${dS}/{dt}=\gamma iP$. Patients with mild to moderate disorders and severe disorders require, respectively, $k/r$ and ${\theta k}/r$ services (on average) before recovering; thus, when $M$ and $S$ are near to zero, all patients presenting for care receive immediate and effective treatment (so that unmet need for services, $M+S$, remains near zero indefinitely) when $C\geq{k\left( 1-\gamma\right)iP}/r+{\theta k\gamma iP}/r={kiP\left( 1-\gamma+\theta\gamma\right)}/r$.

*2.2. Determining equilibrium stability*

The local stability of any particular equilibrium $\left( M^{*},S^{*} \right)$ can be determined from the eigenvalues of the Jacobian matrix

$$J=\left( \begin{matrix} \frac{\partial\left( {dM}/{dt} \right)}{\partial M} & \frac{\partial\left( {dM}/{dt} \right)}{\partial S} \\ \frac{\partial\left( {dS}/{dt} \right)}{\partial M} & \frac{\partial\left( {dS}/{dt} \right)}{\partial S} \end{matrix} \right)=\left( \begin{matrix} -\left( i-\gamma i+v+\mu+u \right)-\frac{r\theta kSC}{\left( kM+\theta kS \right)^{2}} & \gamma i-i+\frac{r\theta kMC}{\left( kM+\theta kS \right)^{2}} \\ v-\gamma i+\frac{rkSC}{\left( kM+\theta kS \right)^{2}} & -\left( \gamma i+\eta\mu+u \right)-\frac{rkMC}{\left( kM+\theta kS \right)^{2}} \end{matrix} \right)\text{,}$$

evaluated at the equilibrium. Where all eigenvalues are negative, the equilibrium is locally stable (i.e., the system will tend to return to the equilibrium when disturbed, provided the disturbance is relatively small); otherwise, the equilibrium is unstable (in this case, the system will move away from the equilibrium when disturbed) (see Otto and Day, 2007, Chapter 8).

According to the Routh-Hurwitz conditions (see Otto and Day, 2007, pp. 309−312), the eigenvalues of $J$ will be negative only if the trace of $J$ is negative and the determinant of $J$ is positive, i.e.,

$$\text{Tr}\left( J \right)=\frac{\partial\left( {dM}/{dt} \right)}{\partial M}+\frac{\partial\left( {dS}/{dt} \right)}{\partial S}=-\left( i-\gamma i+v+\mu+u \right)-\frac{r\theta kSC}{\left( kM+\theta kS \right)^{2}}-\left( \gamma i+\eta\mu+u \right)-\frac{rkMC}{\left( kM+\theta kS \right)^{2}}=-\left( i+v+\mu+\eta\mu+2u \right)-\frac{rC}{kM+\theta kS}<0$$

$$\text{Det}\left( J \right)=\frac{\partial\left( {dM}/{dt} \right)}{\partial M}\cdot\frac{\partial\left( {dS}/{dt} \right)}{\partial S}-\frac{\partial\left( {dM}/{dt} \right)}{\partial S}\cdot\frac{\partial\left( {dS}/{dt} \right)}{\partial M}=\left[ -\left( i-\gamma i+v+\mu+u \right)-\frac{r\theta kSC}{\left( kM+\theta kS \right)^{2}} \right]\left[ -\left( \gamma i+\eta\mu+u \right)-\frac{rkMC}{\left( kM+\theta kS \right)^{2}} \right]-\left[ \gamma i-i+\frac{r\theta kMC}{\left( kM+\theta kS \right)^{2}} \right]\left[ v-\gamma i+\frac{rkSC}{\left( kM+\theta kS \right)^{2}} \right]>0\text{.}$$

The dynamic model parameters are all non-negative, so $\text{Tr}\left( J \right)<0$ whenever $M$ and $S$ are positive (which is true for the equilibria considered here). Thus, an equilibrium $\left( M^{*},S^{*} \right)$ will be locally stable when

$$\left[ -\left( i-\gamma i+v+\mu+u \right)-\frac{r\theta kS^{*}C}{\left( kM^{*}+\theta kS^{*} \right)^{2}} \right]\left[ -\left( \gamma i+\eta\mu+u \right)-\frac{rkM^{*}C}{\left( kM^{*}+\theta kS^{*} \right)^{2}} \right]-\left[ \gamma i-i+\frac{r\theta kM^{*}C}{\left( kM^{*}+\theta kS^{*} \right)^{2}} \right]\left[ v-\gamma i+\frac{rkS^{*}C}{\left( kM^{*}+\theta kS^{*} \right)^{2}} \right]>0\text{.}$$

*2.3. Number of patients treated per year*

The total numbers of services provided to people with mild to moderate and severe mental disorders per year are ${kMC}/\left( kM+\theta kS \right)$ and ${\theta kSC}/\left( kM+\theta kS \right)$, respectively (section 2.1 above). Patients with mild to moderate disorders each require $k$ services, so the number of people with mild to moderate disorders treated per year is equal to $\left[ {kMC}/\left( kM+\theta kS \right) \right]/k={MC}/\left( kM+\theta kS \right)$. Similarly, the number of patients with severe disorders treated per year is $\left[ {\theta kSC}/\left( kM+\theta kS \right) \right]/\left( \theta k \right)={SC}/\left( kM+\theta kS \right)$ (where each patient requires $\theta k$ services). Thus, the total number of patients treated per year is

$$\frac{MC}{kM+\theta kS}+\frac{SC}{kM+\theta kS}=\frac{C\left( M+S \right)}{k\left( M+\theta S \right)}\text{.}$$

*2.4. Proportion of people engaged with services who have a severe disorder*

Applying the quotient rule, the rate of change in the proportion of people engaged with specialised services who have a severe disorder is

$$\frac{d\left( \frac{S}{M+S} \right)}{dt}=\frac{\left( M+S \right)\frac{dS}{dt}-S\frac{d\left( M+S \right)}{dt}}{\left( M+S \right)^{2}}=\frac{\left( M+S \right)\left[ \gamma i\left( P-M-S \right)+vM-S\left( \eta\mu+u \right)-\frac{rSC}{k\left( M+\theta S \right)} \right]}{\left( M+S \right)^{2}}-\frac{S\left[ i\left( P-M-S \right)-M\left( \mu+u \right)-S\left( \eta\mu+u \right)-\frac{rC\left( M+S \right)}{k\left( M+\theta S \right)} \right]}{\left( M+S \right)^{2}}=\frac{i\left( P-M-S \right)\left[ M\gamma-S\left( 1-\gamma\right) \right]+vM\left( M+S \right)-MS\mu\left( \eta-1 \right)}{\left( M+S \right)^{2}}$$

In the Discussion section of the paper, we note that as the number of South Australian children and adolescents engaged with tertiary-level mental health services declines in response to an increase in services capacity, the proportion of children and adolescents engaged with services who have severe disorders also declines. Panel A in figure S2 shows that as specialised services capacity increases (moving along the upper, stable part of the curve), the equilibrium proportion of children and adolescents with severe disorders engaged with services, ${S^{*}}/\left( M^{*}+S^{*} \right)$, decreases gradually until capacity reaches the critical value $C_{b}$ (equal to 71616 services per year; see figure 4 of the paper). The results in panel B of figure S2 show that, past this point, as the number of children and adolescents engaged with tertiary-level services declines abruptly towards zero, the proportion of children and adolescents with severe disorders declines as well (the system either does not leave or eventually moves into the blue part of the plot, where the derivate ${d\left[ S/\left( M+S \right) \right]}/{dt}$ is negative).

References

Otto, S. P., Day, T., 2007. A biologist’s guide to mathematical modeling in ecology and evolution. Princeton University Press, Princeton.


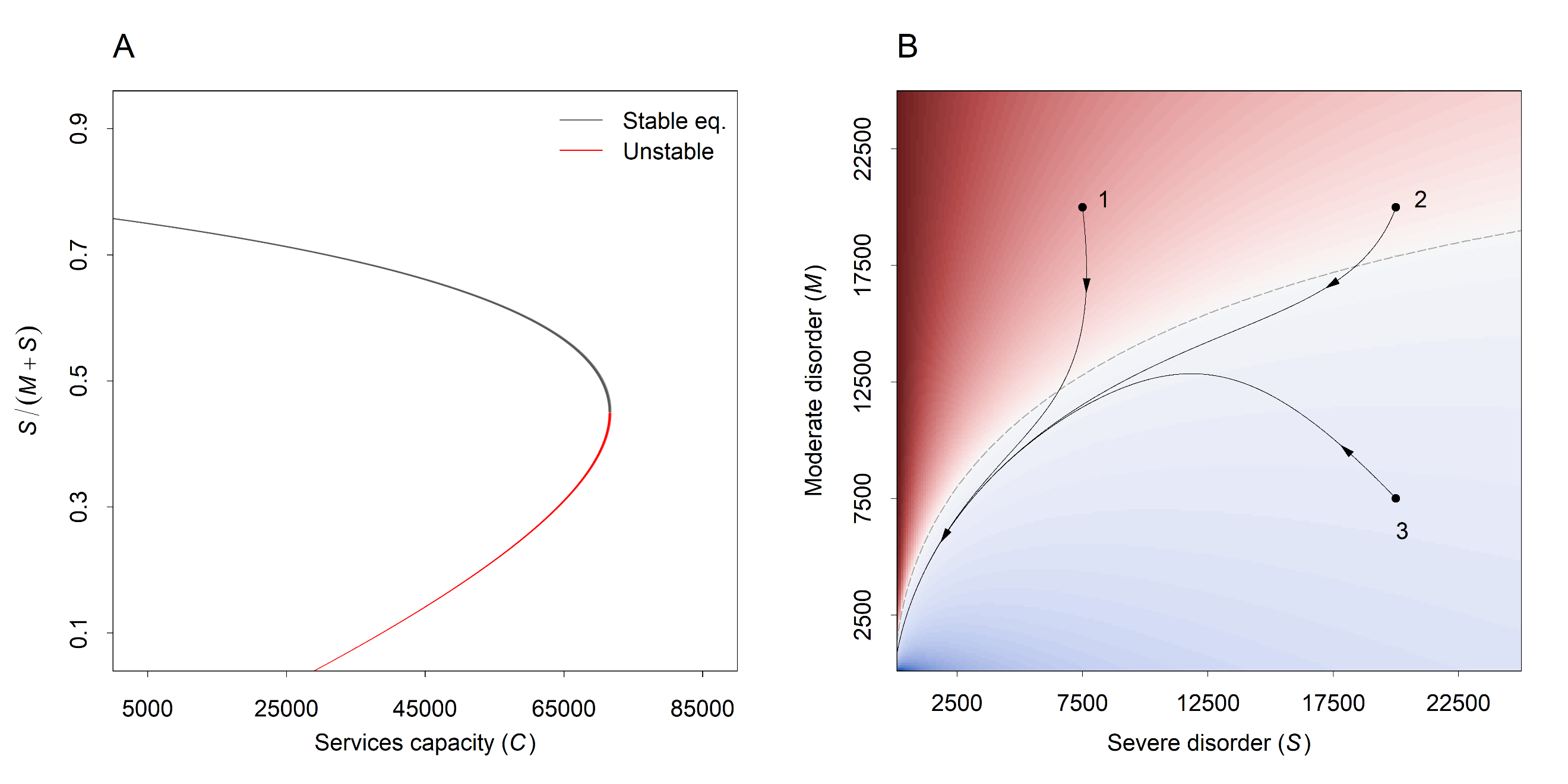


Figure S2. A) Equilibrium proportions of South Australian children and adolescents engaged with tertiary-level mental health services who have a severe disorder over a range of values of the services capacity parameter $C$. Each line was generated from one of 100 randomly selected parameter vectors $(\phi$) sampled in the Markov chain Monte Carlo (MCMC) analysis. B) Derivative of the proportion of children and adolescents engaged with tertiary-level services who have a severe disorder as a function of the numbers of children and adolescents with moderate and severe disorders engaged with services. The derivative is negative in the blue region of the plot and positive in the red region; darker colours correspond to more rapid change. The dashed grey line is the zero isocline (the derivative is equal to zero at all points along this line). System trajectories are shown for three different initial states, labelled 1−3, assuming a constant services capacity of 10^5^ services per year (note that although the trajectories depend on services capacity, the derivative does not, so the distribution of colours would not change under different capacity assumptions). Because services capacity is greater than the critical value $C_{b}$ (equal to 71616 services per year; see figure 4, panel C of the paper), the number of children and adolescents engaged with tertiary-level services declines to near zero in all cases. Note that all three trajectories approach this stable state of no unmet need for services in the blue region of the plot, so the proportion of children and adolescents engaged with services who have a severe disorder declines (at least eventually) with declining unmet need for specialised care. Both plots were generated assuming a constant total population of 365965 children and adolescents (the estimated number of South Australian residents aged 0−17 years in 2017); for the plot in panel B, we assumed median estimates for the per capita rates $i$ and $v$ derived from the MCMC analysis (see figure 4 of the paper).

Supplementary appendix 3

Markov chain Monte Carlo (MCMC) simulation methods

Data on the prevalence of moderate and severe mental health problems among South Australian children and adolescents (aged 0−17 years) were derived from Segal et al. (2018, their Table 1, where $M$ in our model corresponds to Group B + Group C and $S$ corresponds to Group A). Prevalence estimates for 2017−2019 were calculated assuming that the age-specific proportions of children and adolescents with moderate and severe psychiatric conditions reported by Segal et al. (2018) for 2017 were stable over this period (consistent with long-term trends in the prevalence of child and adolescent mental disorders in Australia; Lawrence et al., 2015, p. 138); thus, changes in prevalence over time were assumed to result from the changing age structure of the population only. Data from the second Australian Child and Adolescent Survey of Mental Health and Wellbeing (see Lawrence et al., 2015, their Table 8-4) indicate that 8.2% of parents and carers of children and adolescents with moderate mental health problems do not perceive a need for treatment. We therefore multiplied all estimates of the prevalence of moderate psychiatric conditions by 0.918 to accommodate the fact that these children and adolescents would not be expected to engage with specialised services (note that all parents and carers of children and adolescents with severe mental health problems reported perceiving a need for help).

Posterior simulation was performed using Stan ver. 2.21.2 (Carpenter et al., 2017). Prior distributions for the dynamic model parameters ($i$, $v$, and the initial proportions of children and adolescents with moderate and severe psychiatric conditions) are in Table 2 of the paper. We specified exponential prior distributions with means equal to 10^9^ for the beta distribution parameters $\alpha_{j}$ (see Methods section of the paper). Four Markov chains, each initialised with parameter values sampled from the joint prior distribution, were run in parallel for 4000 iterations; we used the final 2000 iterations from each chain (8000 samples in total) for posterior inference (the initial half of each chain was discarded as warmup). Potential scale reduction factors ($\hat{R}$) calculated for all parameters were less than 1.01, indicating approximate convergence to the posterior distribution (see Gelman et al., 2014, pp. 281‒286). Trace plots for the dynamic model parameters are shown in figure S3.


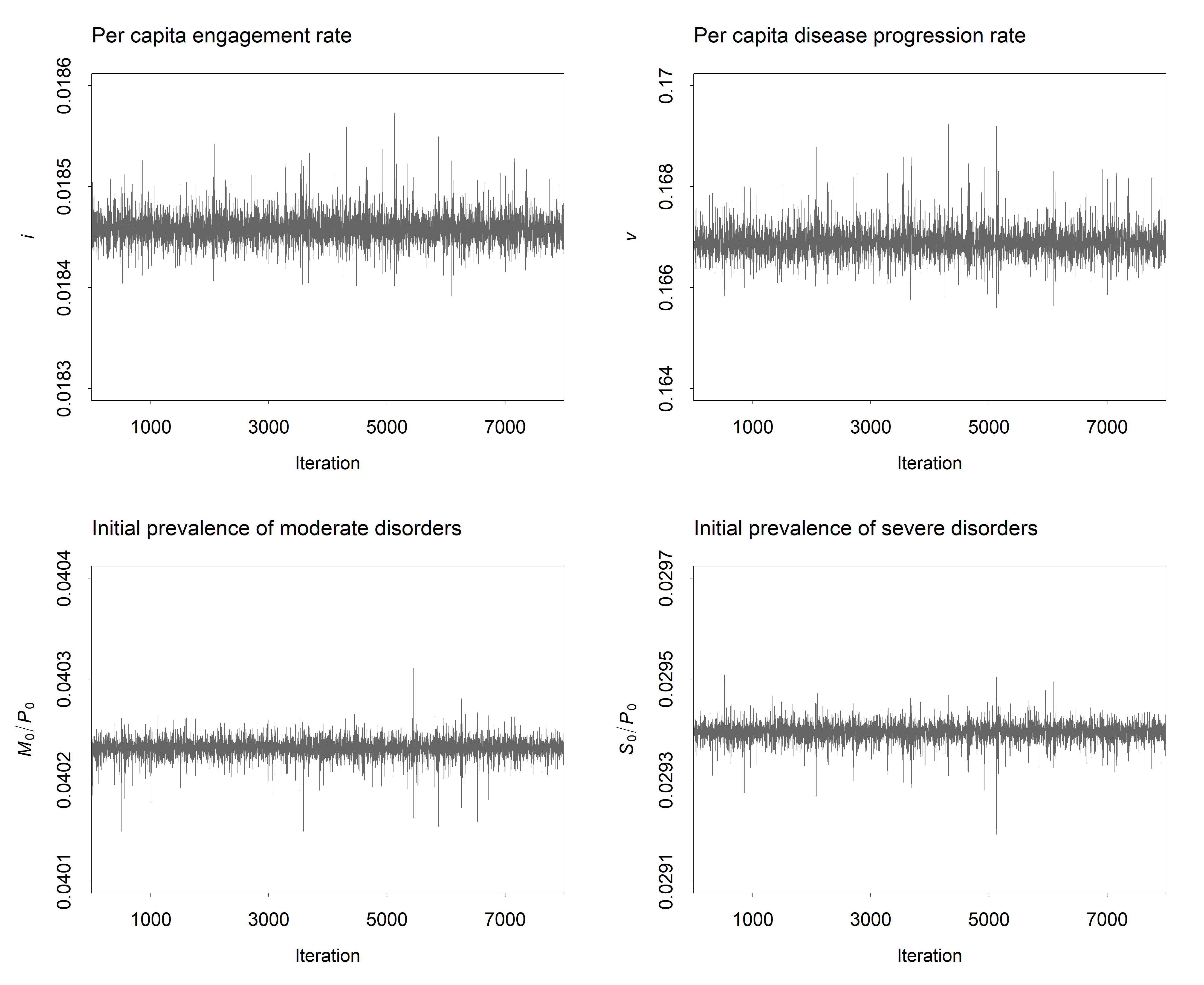


Figure S3. Trace plots for the per capita rate at which children and adolescents engage with specialised services ($i$), the per capita rate of progression from moderate to severe disorders ($v$), the initial prevalence of severe disorders (i.e., at the start of 2017), and the initial prevalence of moderate disorders. Post-warmup samples from the four separate Markov chains are concatenated in each plot, i.e., samples 1‒2000 are from the first chain, samples 2001‒4000 are from the second chain, and so on.

References

Carpenter, B., Gelman, A., Hoffman, M. D., Lee, D., Goodrich, B., Betancourt, M., Brubaker, M. A., Guo, J., Li, P., Riddell, A., 2017. Stan: a probabilistic programming language. J. Stat. Softw. 76 (1), 1‒32.

Gelman, A., Carlin, J. B., Stern, H. S., Dunson, D. B., Vehtari, A., Rubin, D. B., 2014. Bayesian data analysis. CRC Press, Boca Raton.

Lawrence, D., Johnson, S., Hafekost, J., Boterhoven de Haan, Sawyer, M., Ainley, J., Zubrick, S. R., 2015. The mental health of children and adolescents. Report on the second Australian Child and Adolescent Survey of Mental Health and Wellbeing. Department of Health, Canberra.

Segal, L., Guy, S., Leach, M., Groves, A., Turnbull, C., Furber, G., 2018. A needs-based workforce model to deliver tertiary-level community mental health care for distressed infants, children, and adolescents in South Australia: a mixed-methods study. Lancet Public Health 3, e296‒303.

Supplementary appendix 4

Effect of increasing services capacity on the time required to transition between alternative system states


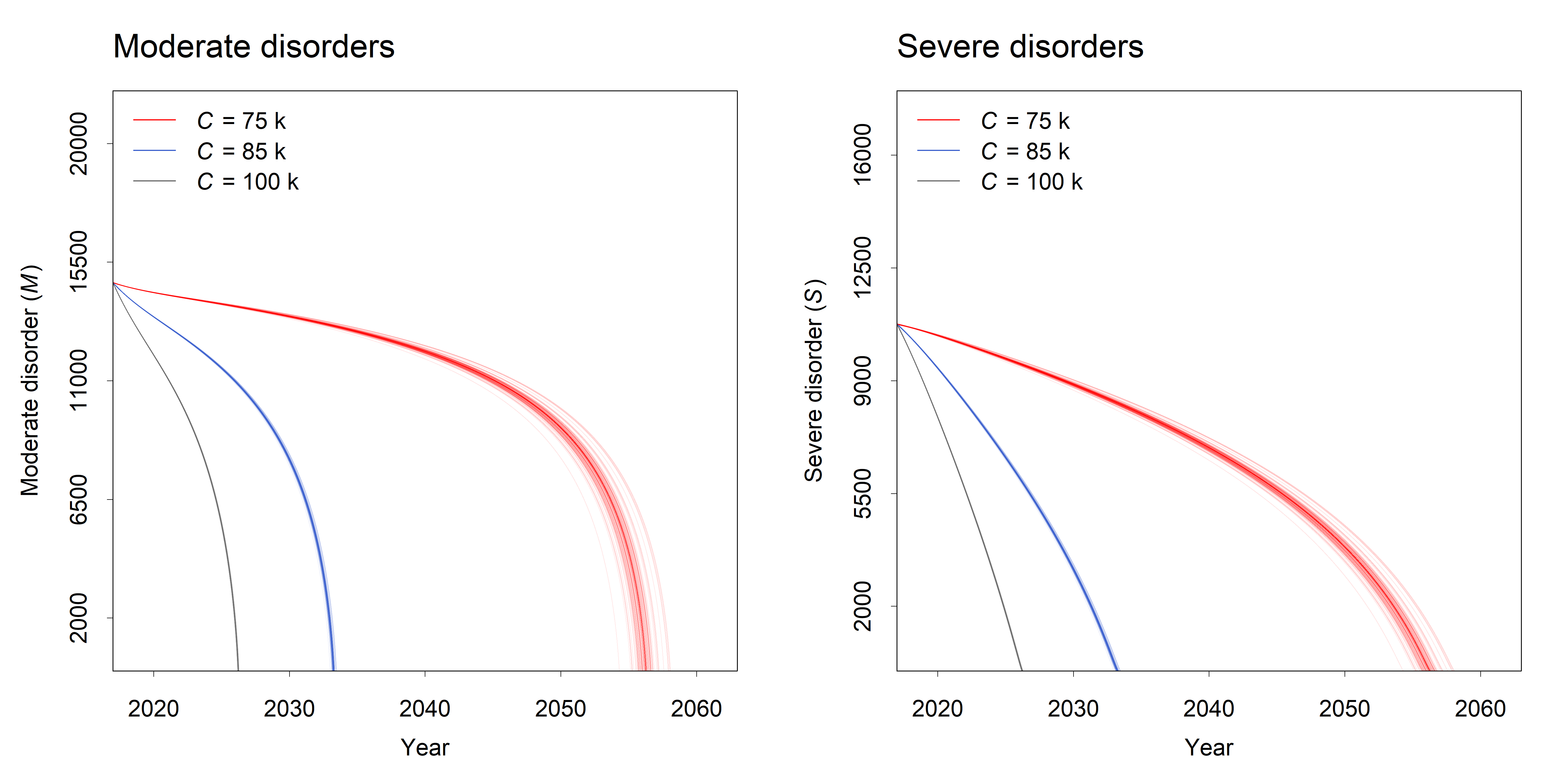


Figure S4. Numbers of South Australian children and adolescents (aged 0−17 years) with moderate and severe psychiatric conditions engaged with tertiary-level services. All simulations were run assuming a constant population (365965, the estimate for 2017). Lighter lines are based on 100 randomly selected parameter vectors $(\phi$) sampled in the Markov chain Monte Carlo (MCMC) analysis (see Methods section of the paper and Supplementary appendix 3); the heavy lines were generated assuming median estimates for the per capita rates $i$ and $v$, the initial prevalence of severe disorders (${S_{0}}/{P_{0}}$), and the initial prevalence of moderate disorders (${M_{0}}/{P_{0}}$). Note that as services capacity ($C$) increases above the threshold value $C_{b}$, equal to 71616 services per year (see Methods section of the paper), the time required for the number of children and adolescents engaged with services to decline to near zero decreases significantly.
